# Tackling the challenge of peer learning in hybrid and online universities

**DOI:** 10.1007/s10639-022-11397-7

**Published:** 2022-10-20

**Authors:** Adrián Mendieta-Aragón, Raquel Arguedas-Sanz, Luis Manuel Ruiz-Gómez, Julio Navío-Marco

**Affiliations:** 1 Department of Economic Analysis, National University of Distance Education (UNED), Madrid, Spain; 2 Facultad de Económicas, Universidad Nacional de Educación a Distancia, Paseo Senda del Rey, 11, 28040 Madrid, Spain; 3 Department of Business Economics and Accounting, National University of Distance Education (UNED), Madrid, Spain; 4 Department of Business Organization, National University of Distance Education (UNED), Madrid, Spain

**Keywords:** Peer learning, Blended learning, Learner-generated content, Online teaching, Hybrid university

## Abstract

Peer learning is not fully developed or researched in online and hybrid higher education. This research analyses a peer learning experience in the asynchronous part of hybrid teaching, in one of the largest blended universities in Europe, promoting students to act as teachers of their peers, by preparing digital content (videos) for the course. This article studies whether there are behaviour patterns and different perceptions associated between students who act as teachers, and those who only act as students. The results indicate, among other findings, that students demand this type of activities, and value them very positively. Specifically, the “teachers” consider that this activity increases their motivation for the subject and their performance; they also consider that it significantly improves their creativity and communication skills, and they would definitely participate in the project again. The assessment of the students who merely view the materials is also very positive, and they prefer a learning method through classmate videos than the traditional learning method with printed materials. The research is also a boost to finding ways to promote learning among equals in non-classroom teaching in digital environments.

## Introduction

The Covid-19 pandemic has led to a huge expansion in hybrid (blended, semi-remote, mixed) and online learning, arousing considerable interest in better understanding its peculiarities and possibilities, lending particular momentum to research in this regard (Aretio, 2021; Chandra & Palvia, 2021; Okoye et al., 2021; Zhou et al., 2022). Alongside the discussion as to the virtues of such types of teaching, questions arise as to how online teaching can support many of the established benefits of an in-person learning environment, including student participation and peer interaction (Dunford and Miller, 2018; McDonald et al., 2021; Muir et al., 2019; Tang et al., 2022).

It becomes particularly important to guarantee learner participation (Kaur, 2013) and to promote student self-regulatory processes in such remote environments (Pool et al., 2017), fostering the development of their own learning environments, networks and communities. Boelens et al. (2017) suggest giving them greater responsibility, promoting active involvement and introducing a problem-based focus.

Rasheed et al. (2021), in their recent review of the challenges of blended learning, and in particular the online element, consider that peer learning regulation strategies have not yet been provided. They stress that online peer learning is one of the main challenges in hybrid learning instruction, and is closely linked to the involvement of students and co-production of knowledge (Cullen, 2020).

This research contributes to an attempt to mitigate such shortcomings, proposing a peer learning experience in the asynchronous (digital) part of hybrid teaching. The proposed learning method encourages university students to act as the teachers of their fellow learners, preparing digital material (videos in this case) to explain content and resolve new problems and examples of the subject. This serves to generate a stock of problems solved, which helps their fellow students in their learning, fosters the creation of a learning community, and serves to drive the motivation and engagement of the students themselves, as they “teach” their peers. Furthermore, as this involves the creation of videos from which the other students learn, they must be fully familiar with the contents of the subject to be delivered, and, as they are dealing with other students, this mitigates the isolation that they may feel in such educational settings.

The specific research question raised in this paper is whether there are behavioural patterns and different associated perceptions among students who act as teachers, and those who act only as students. The research thus helps to establish a better understanding of peer learning mechanisms, and the creation of digital content by learners in remote settings. It furthermore constitutes an invitation to disseminate and promote this approach.

The article is structured as follows: after this introduction, Section 2 presents the theoretical framework, while Sections 3 and 4 contain the description of the sample, the methodology, the empirical analysis and the results. Lastly, the article ends with the conclusions, which likewise include the limitations and future avenues of research.

## Theoretical framework

In this section, we will explore in greater depth the underlying principles of peer learning, and, in particular, research into this phenomenon in hybrid and online teaching environments, as well as student-generated content, above all in the digital world.

### Peer learning in hybrid and online teaching

Over the years, many researchers have studied the concept of peer learning (Gamlath, 2021; Ion et al., 2016; Latifi et al., 2021). One of the pioneers was Topping (1996), who defined peer learning as the process which takes place when people with similar background, but who are not educators, work together and teach one another in order to understand certain topics. Mazur (1997) developed the concept and popularised it in his teaching at Harvard in the 1990s. In general, peer learning refers to the capacity among students to work with their fellows and support one another in learning (Lim et al., 2020).

Peer instruction is an active learning strategy which underpins learning, promotes a deeper engagement and also fosters conceptual understanding through instruction outside the classroom (Mazur, 1997; Nerantzi, 2020; Schell & Mazur, 2015). Peer learning helps to create an audience, drives students’ knowledge as to their own strengths and weaknesses, and offers opportunities for collaborative learning (Xiao & Lucking, 2008). Furthermore, as indicated by Chandra and Palvia (2021), by valuing cooperation rather than competition within the group, it helps foster mutual respect with regard to the gender, age and cultural background of the participants.

In hybrid and online settings, where interactions between students and the teaching staff and content may be made more difficult by the involvement of technology, one of the major challenges is to maintain the benefits of interaction and learning among students (Solórzano-García & Navío-Marco, 2019; Nerantzi, 2020) is of the opinion that although its potential is recognised for in-person courses, it is likewise of use for hybrid and completely online settings. The classroom may, in this case, be a live or synchronous online session bringing together the students and teacher, while the asynchronous activities, or those followed at their own pace, could include peer learning. In considering the use of digital environments, one may refer to “digital peer learning” (Sannino et al., 2021), to emphasise the role of the technology used.

This type of educational strategy incorporates the postulates of constructivist theories, and in particular the social constructivism of Vygotsky (1962; 1978), which considers that learning takes place when individuals participate in social activities such as interaction and collaboration. It is through social interaction that individuals are able to develop cooperation and mutual respect until they achieve autonomy (Barvinski et al., 2019). The learning communities to which students belong form a relevant part of this environment. These theoretical fundamentals may therefore be interlinked with the concepts of Communities of Practice (CoP), which have been used to understand the social aspects of training in learning communities to create bonds and foster learner interaction (Bozkurt & Keefer, 2017; Wenger, 1998). Meanwhile, Contributing Student Pedagogy, CSP (Collis & Moonen, 2005; Falkner & Falkner, 2012) emphasises the role of engaging students in the educational process, by adopting an active role as co-creator of resources for learning, and the exchange of content. Ultimately, one may establish a relationship with learning ecologies, understood as the set of contexts to be found in physical or virtual settings, which offer opportunities for learning, with each context made up of a particular configuration of activities, resources, relationships and the interactions that emerge from them (Barron, 2006; Peters et al., 2021; Sangrá et al., 2019). Within such contexts, there must be a place for peer interactions and student-generated content.

### Student-recorded videos

One strand of research focuses on Learner-Generated Digital Media (LGDM), where such digital content developed by the students demonstrates their learning (Reyna et al., 2021; Navio-Marco et al., 2022). This is an original focus to improve research capabilities and active learning (Anuradha & Rengaraj, 2017; Hoban et al., 2015), but has not yet been the subject of much analysis, particular regarding its use in higher education (Hakkarainen, 2009; Potter & McDougall, 2017; Reyna & Meier, 2018). Research in this regard focuses in general on in-person studies, and in particular the flipped classroom (Engin, 2014; González Fernández & Huerta Gaytán, 2019). It may be seen that in producing digital content, students show a greater level of refinement of thought and in-depth analysis, which in many cases results in greater academic performance, and has a positive effect on learning outcomes (Bates et al., 2012; Bryson & Hand, 2007; Fellenz 2004; Hardy et al., 2014).

In particular, the production of videos involves greater understanding and higher levels of cognitive processing in order to create contents, teach and evaluate (Engin, 2014; Gerbaudo et al., 2021).

## Methodology

To provide a response to our research question, 78 engineering students on the course Economics and Business volunteered to take part in the project over the academic years 2020/21 and 2021/22 in the different roles of the learning community: Student-Teacher (those who recorded videos to support the learning of their peers) and Student-Learner (those who simply used their fellow students’ videos in their studies). The teaching team distributed topics with problems for application in the field of economics. Then, the students proposed examples of resolved problems, explaining their approach and solution through videos. The videos were first supervised by the teaching team, and then made available to the students as a bank of problems solved, while the students who produce them cleared up queries in the forums.

Once the project had ended, the participants completed a survey to ascertain their sociological and professional profile, as well as their opinion on the functioning of the activity. The opinion responses were structured by means of a Likert scale from 1 to 5, with 1 indicating that the respondent did not agree at all, and 5 that they completely agreed with the proposition.

In accordance with the purpose of the analysis and the nature of the data, univariate descriptive techniques were first used to ascertain the sociological and professional characteristics of the students involved in the project, as well as the overall evaluation of the activity. Bivariate inferential statistical techniques are then used to identify possible differences between the participants in the learning communities, in terms of their evaluation of and satisfaction with the activity.

The data analysis employs the Kruskal-Wallis or H test (Kruskal & Wallis, 1952). This test is extensively used in educational projects to compare and contrast the hypothesis that the different sample groups were drawn from the same population (Atik & Yalçin, 2010). This test was chosen for two main reasons: it is a non-parametric test which can be used to conduct an analysis with a smaller sample size (Grech & Calleja, 2018); and it is an effective test to analyse items on a Likert scale of independent groups (Nipa & Kermanshachi, 2020).

The Kruskal-Wallis H statistic was obtained as:

1 $$H= \frac{12}{N(N+1)}\sum _{i=1}^{k}\frac{{R}_{i}^{2}}{{n}_{i}}-3(N-1)$$

where *N* represents the total observations; *k* indicates the number of groups; *R* indicates the range of the group; and *n*_*i*_ the number of observations of group *i.*

When the Kruskal-Wallis test considers that there are significantly different effects in categories with three or more groups, a *post-hoc* comparison will be conducted between pairs by means of the Dunn test, to precisely determine which groups are different. To avoid an increase in the type I error, the level of significance is adjusted by means of the Bonferroni method, which makes the analysis more robust, as it is one of the strictest adjustment methods.

## Empirical analysis and results

### Descriptive analysis

The information gathered by means of the questionnaires was processed using STATA v.17 software. The Alpha-Cronbach test confirms the reliability and consistency of the questionnaire measurements (α = 0.9530).

Table 1 shows the sociological and professional profile of the students participating in the project. The results highlight the greater presence of men, with the majority age range being between 30 and 45 years. One of the main advantages of remote universities is the greater flexibility that they offer students in combining their university studies with a job (part-time or full-time). This characteristic of semi-remote students is clearly reflected in our sample, with 80.7% of students combining their studies with full-time employment. Lastly, the distribution of participants across the learning communities reveals 71.8% in the role of Student-Learner, and 28.2% in the role of Student-Teacher.

**Table 1:** Sociological-professional profile of the participants

	Frequency (%)
Gender	
Man	61 (78.2%)
Woman	17 (21.8%)
Age	
[18–30 years)	22 (28.2%)
[30–45 years)	38 (48.7%)
[45 years and over)	18 (23.1%)
Employment Situation	
Part-time employment	8 (10.3%)
Full-time employment	63 (80.7%)
Not working	7 (9.0%)
Role	
Student-Learner	56 (71.8%)
Student-Teacher	22 (28.2%)

Source: Produced by the author

**Fig. 1:**
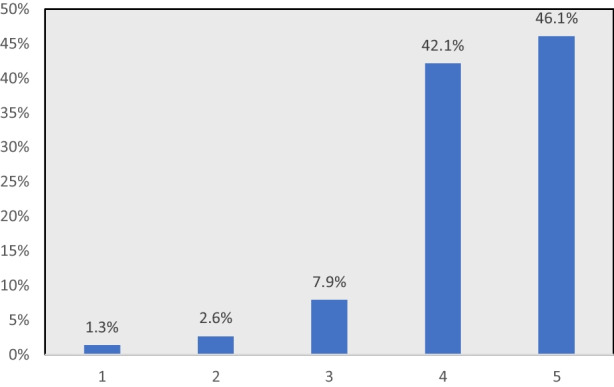
Overall level of satisfaction with the project. Source: Produced by the author

Figure 1 shows the overall satisfaction with the project to prepare and assimilate the subject content. The graph shows the high rating given by the students to the creation of videos to pass the subject, indicating that there is a demand for this type of activity among the students. In quantitative terms, 88.2% of the respondents gave a high score of 4–5, while just 3.9% of participants gave a low score (1–2).

### Inferential analysis

In this section, we present the results of the bivariate analysis using the H statistic of the Kruskal-Wallis test to detect possible statistically significant differences among the different student profiles and the mean evaluation of the items presented.

Table 2 shows the mean of each category in each item and the H statistic, from the perspective of the student creating content in the learning community. The empirical results show that the proposed activity increases motivation for the subject (item 1a) and student performance (item 1b). Furthermore, the students in the Student-Teacher role consider that there is a significant improvement in their creativity (item 1d) and communication skills (item 1f), which is an important aspect to convey knowledge among peers.

Subject-matter creation enables the students to improve their capacity to fix content in the learning process (item 2a) and to summarise the fundamental concepts of the exercise (item 2b). Regarding this first aspect, the Kruskal-Wallis and Dunn tests detect significant differences between students aged 18 to 30 and those over 45 (Dunn *p*-value = 0.02). Therefore, creating videos has a greater effect in helping to fix content in the learning process within the group of individuals belonging to Generations Z and Y, considered to be digital natives. Furthermore, recording the videos facilitates active learning among the students (item 3d), particularly among the younger age groups (Dunn *p*-value = 0.034).

Meanwhile, a high average score is given to the inclusion of the recorded videos in a repository for future students of the subject (item 5), which fosters active learning through the creation of virtual learning forums developed by the students themselves. It also drives collective cooperation in subsequent academic years. However, the content creators prove more averse to the videos recorded being distributed beyond this virtual learning forum, in particular on platforms such as Facebook, Twitter or Instagram. Presentation on YouTube obtained a more favourable response.

With regard to the stage involving the greatest effort for the participants in performing the activities, we would highlight the stages corresponding to the preparation of content, such as structuring and summarisation (item 10b) and content generation (item 10c). In this latter regard, significantly greater effort was seen in the case of students working full time, compared with students who are not in work (Dunn *p-*value = 0.0043), who reveal greater limitations on their study time.

Lastly, the generation of content by those students in the role of teacher was highly satisfactory, with an average score of 4.83 out of 5 indicating that they would take part in the project again (item 11). It also demonstrates that the creation of collaborative digital environments in a hybrid university strengthens skills and the learning process of the students playing an active part in the peer teaching communities.

**Table 2:** Student-Teacher activities: Inferential analysis

	Item	GENDER			AGE				EMPLOYMENT SITUATION				ROLE			TOTAL
		Man	Woman	H-test	[18–30)	[30–45)	[45-over)	H-test	Part time	Full time	Not working	H-test	Student- Learner	Student-Teacher	H-test	
1	The creation of digital content:															
	a. Increases my motivation	4.33	4.75	2.67	4.50	4.44	4.33	0.50	4.00	4.52	4.25	0.73	4.29	4.50	0.18	4.25
	b. Increases my performance	4.33	4.63	1.25	4.40	4.38	4.67	0.31	4.00	4.43	4.50	0.33	4.14	4.50	0.99	4.41
	c. Increases my responsibility for learning	4.38	4.38	0.09	4.40	4.56	3.33	1.38	3.50	4.43	4.50	0.42	4.57	4.32	0.08	4.38
	d. Increases creativity	4.29	4.50	0.24	4.40	4.50	3.33	2.18	4.00	4.30	4.75	2.02	3.57	4.59	4.55**	4.34
	e. Builds useful and significant knowledge	4.24	4.50	0.24	4.40	4.44	3.33	4.08	3.50	4.39	4.25	3.08	4.14	4.36	0.42	4.31
	f. Develops my communication and knowledge transfer skills	4.33	4.50	0.66	4.20	4.56	4.00	2.93	3.00	4.52	4.25	6.21**	3.86	4.55	3.72*	4.38
2	The capacity to produce digital content helped me:															
	a. Fix content in the learning process	4.30	4.88	3.49*	4.78	4.44	3.67	5.46*	5.00	4.48	4.25	1.60	4.33	4.50	0.29	4.46
	b. Summarise the key aspectsof the problem	4.40	4.63	0.55	4.67	4.44	4.00	3.17	4.00	4.48	4.50	0.82	4.50	4.45	0.00	4.46
	c. Improve oral presentational skills about an economic topic	4.35	4.38	0.09	4.22	4.50	4.00	1.04	2.00	4.39	4.75	4.11	4.50	4.32	0.009	4.36
	d. Improve results in evaluation of the subject	4.45	4.25	0.20	4.56	4.31	4.33	1.02	3.00	4.48	4.25	3.62	4.67	4.32	1.11	4.39
3	Involvement in the project allowed me to:															
	a. Trust in my skills to face new learning challenges	3.95	3.56	0.76	4.00	3.81	3.33	1.18	2.33	3.96	4.25	6.66**	3.50	3.95	0.95	3.83
	b. Improve my problem-solving capacity	4.00	3.89	0.01	4.09	4.00	3.33	3.11	3.00	4.04	4.25	4.02	3.88	4.00	0.01	3.97
	c. Develop information search and management skills	4.14	3.75	0.72	4.10	4.06	3.67	0.79	3.00	4.09	4.25	1.98	4.14	4.00	0.05	4.03
4	The skills acquired by producing and recording the video allowed me to:															
	a. Improve my ability to analyse and summarise	3.95	4.00	0.01	4.11	3.94	3.67	0.71	3.00	4.00	4.00	1.56	4.17	3.91	0.51	3.96
	b. Improve how I manage information	4.05	4.13	0.07	4.33	4.00	3.67	2.36	4.00	4.09	4.00	0.06	4.33	4.00	0.95	4.07
	c. Increase my autonomy	3.85	3.88	0.01	4.22	3.81	3.00	1.66	2.00	3.83	4.50	3.03	4.50	3.68	1.99	3.86
	d. Foster active learning	4.10	4.50	1.80	4.44	4.25	3.33	5.25*	4.00	4.17	4.50	0.86	4.17	4.23	0.03	4.21
	e. Improve oral communication	4.20	4.00	0.29	4.33	4.00	4.33	1.19	3.00	4.13	4.50	2.56	4.50	4.05	1.35	4.14
5	I believe that content production fosters skills focused on employability	4.05	3.89	0.08	4.18	3.88	4.00	0.74	4.00	3.87	4.75	3.66	4.13	3.95	0.17	4.00
6	In favour of including the video in a repository for the virtual subject course	4.80	4.63	0.09	4.56	4.81	5.00	0.80	2.00	4.87	4.75	6.59**	4.83	4.73	0.01	4.75
7	In favour of sharing the video recorded with:															
	a. Fellow students studying the subject this academic year	3.81	3.29	1.36	4.11	3.44	3.67	0.95	4.00	3.55	4.25	0.44	3.50	3.73	0.54	3.68
	b. Fellow students studying the subject in future academic years	4.38	4.25	0.03	4.20	4.56	3.67	0.69	2.00	4.52	4.50	6.38**	3.57	4.59	6.06**	4.34
	c. Students on the same degree course	3.48	2.86	0.96	3.44	3.38	2.67	0.52	3.00	3.18	4.25	1.80	3.33	3.32	0.05	3.32
	d. Students enrolled at the UNED	3.62	3.63	0.00	3.10	4.06	3.00	2.52	1.00	3.74	4.25	5.42*	3.00	3.82	1.85	3.62
8	Degree of acceptance of disseminating the digital contents on social media	3.24	3.63	0.49	3.00	3.75	2.33	3.23	2.00	3.39	3.75	2.09	3.29	3.36	0.00	3.34
9	Which social media platforms would they use to share the recording?															
	a. Facebook	2.25	1.00	4.33**	1.13	2.31	2.00	2.87	1.00	2.09	1.25	0.98	1.40	2.05	0.61	1.93
	b. Instagram	2.11	1.86	0.01	2.13	2.00	2.00	0.05	2.00	2.05	2.00	0.56	1.00	2.30	3.18*	2.04
	c. YouTube	3.33	3.25	0.00	3.60	3.25	2.67	1.01	3.00	3.22	4.00	0.40	3.29	3.32	0.04	3.31
	d. Twitter	2.37	1.00	3.94**	1.88	2.07	2.00	0.16	1.00	2.00	2.25	0.90	1.00	2.24	2.55	2.00
	e. LinkedIn	2.63	2.43	0.10	2.56	2.71	2.00	0.49	4.00	2.40	2.75	1.00	2.33	2.65	0.25	2.58
	f. Other	1.81	1.00	2.62	1.43	1.54	2.00	0.46	1.00	1.68	1.00	0.46	1.40	1.61	1.26	1.57
10	Stages of the activity involving the greatest effort:															
	a. Selecting the information	3.45	2.86	1.02	3.22	3.27	3.67	0.41	3.00	3.45	2.50	2.26	3.80	3.18	2.05	3.30
	b. Structuring and summarising content	3.85	3.86	0.12	3.33	4.13	4.00	2.63	4.00	4.05	2.75	2.75	3.60	3.91	0.01	3.85
	c. Generating content	3.35	3.86	1.74	3.00	3.80	3.33	2.59	4.00	3.73	2.00	7.13**	3.40	3.50	0.00	3.48
	d. Learning about the tool to record the digital content	2.40	2.43	0.18	2.56	2.53	1.33	3.74	3.00	2.32	2.75	0.82	2.60	2.36	0.38	2.41
	e. Recording the video	3.00	2.67	0.28	2.89	2.93	3.00	0.03	2.00	2.90	3.25	0.93	3.00	2.90	0.07	2.92
	f. Adapting the content and recording to available time	3.35	2.00	0.35	2.89	3.67	2.33	3.26	2.00	3.50	2.25	3.48	3.00	3.32	0.20	3.26
	g. Producing an educational resource to facilitate collaborative environments	3.40	3.00	0.40	3.22	3.33	3.33	0.01	3.00	3.45	2.50	2.29	3.40	3.27	0.51	3.30
11	Would take part in video content generation again	4.76	5.00	1.70	4.82	4.80	5.00	0.95	4.33	4.86	5.00	1.90	4.57	4.91	1.85	4.83

Note: The values in bold indicate significant differences at 10%(*), 5%(**) and 1%(***). Homoscedasticity of data is assumed

Table 3 presents the results obtained from the mean score given to the activity proposed on the part of students in the learner role. One may emphasise the high degree of satisfaction among students as to the benefits derived from using videos to pass the subject, as well as the creation of collaborative environments during the learning process.

The participants specifically emphasised that the videos recorded by their peers help them to better understand the concepts of the subject (item 12a), and to supplement the teaching materials of the subject, particularly among women. Significant differences are furthermore found in favour of students not only using the videos, but also producing them, thereby facilitating a mutual context of cooperation, benefiting the creation of teaching materials for the learning community. The proposed activity was viewed favourably by the participants, in giving them greater flexibility in their study and the self-regulation of their learning, an essential characteristic in hybrid university teaching. This characteristic was significantly better rated by students in the age range 30 to 45 (Dunn *p-*value = 0.0207). Meanwhile, there was an improvement in the performance in the subject within this same group of students, who represent the majority in hybrid university teaching (Dunn *p-*value = 0.08).

The results also show is that the proposed learning method improves skills that would be more difficult to develop in traditional learning systems (item 14). Similarly, the high mean rating given to item 15 clearly confirms that the students prefer a learning method using videos with content created by their peers, rather than the traditional learning method using printed materials.

Meanwhile, the benefits derived from the use of digital learning resources include better retention of information (item 16a), particularly those also playing the role of teacher. Furthermore, students who work part time reveal a significant improvement in their capacity to retain information, compared with students who do not have a job (Dunn *p-*value = 0.058). Lastly, one of the most relevant results of this study is the high value placed by students on facilitating collaborative learning environments, where information circulates horizontally, generating user networks among their peers. This demonstrates the potential demand for the implementation of this learning method on the part of students themselves.

**Table 3:** Evaluation by Student-Learner: Inferential analysis

	Item	GENDER			AGE				EMPLOYMENT SITUATION				ROLE			TOTAL
		Man	Woman	H-test	[18–30]	[30–45]	[45-over]	H-test	Part time	Full time	Not working	H-test	Student- Learner	Student-Teacher	H-test	
12	The videos recorded by other students helped them to:															
	a. understand the theoretical and practical concepts	4.03	4.35	3.69*	4.00	4.24	3.94	2.10	4.13	4.08	4.33	0.34	4.04	4.29	2.07	4.11
	b. Supplement the teaching materials for the subject	4.03	4.35	3.91**	4.00	4.24	3.94	2.12	4.00	4.10	4.33	0.34	4.02	4.33	3.47*	4.11
	c. Plan their learning better	3.58	3.94	1.89	3.86	3.76	3.18	3.78	3.38	3.68	3.83	0.35	3.55	3.95	2.26	3.66
	d. Give them greater self-regulation of their learning	3.78	3.76	0.07	3.76	4.00	3.29	6.17**	3.38	3.80	4.00	0.88	3.73	3.90	0.57	3.77
	e. Improve their performance in the subject	3.90	3.94	0.91	3.67	4.16	3.65	5.15*	3.75	3.92	4.00	0.26	3.84	4.10	1.23	3.91
13	They believe that they have the digital skills to search for and distinguish useful information	4.22	4.29	0.00	4.29	4.18	4.29	0.27	4.50	4.19	4.33	0.81	4.31	4.05	2.01	4.24
14	The proposed activity allowed them to develop skills that are more difficult to achieve with other traditional methodologies on offer:	3.95	4.00	0.19	4.10	4.13	3.41	4.51	4.13	3.90	4.33	1.07	3.93	4.05	0.44	3.96
15	They found learning through the content videos more effective than printed materials	4.39	4.47	0.93	4.52	4.45	4.18	2.32	4.75	4.35	4.50	1.71	4.35	4.57	2.69	4.41
16	The inclusion of digital learning resources serves to:															
	a. Improve information retention	4.08	4.24	1.39	4.33	4.08	3.94	1.60	4.75	4.05	4.00	6.01**	3.98	4.48	5.78**	4.12
	b. Make the educational content more permanent	3.98	4.18	1.49	4.19	4.03	3.82	0.69	4.38	3.95	4.33	1.14	4.04	4.00	0.11	4.03
	c. Provide feedback about learning	4.10	4.18	0.71	4.43	3.97	4.06	2.80	4.75	4.00	4.50	5.04*	4.11	4.14	0.03	4.12
	d. Reuse and improve the content	4.15	4.29	0.81	4.52	4.05	4.06	3.51	4.75	4.10	4.33	4.05	4.20	4.14	0.00	4.18
17	They see it as important to facilitate models in which information circulates horizontally, generating user networks which create collaborative learning environments	4.41	4.65	4.47**	4.62	4.47	4.24	1.35	4.75	4.42	4.50	1.26	4.38	4.67	1.45	4.46

Note: The values in bold indicate significant differences at 10%(*), 5%(**) and 1% (***). Homoscedasticity of data is assumed

## Conclusion

Digital content production on the part of students, and strategies to facilitate peer learning, are aspects that still have to be developed, and have seen little research in hybrid and online university teaching. This study represents a genuine contribution in establishing the theoretical framework and better understanding the perceptions and profiles of the students who generate such content with the aim of teaching their peers, as well as those students who learn through such digital content.

The students show a demand for this type of activity, which they rate very highly. Specifically, the students acting as “teachers” believe that this activity increases their motivation for the subject and their performance, while likewise feeling that it makes a significant improvement to their creativity and communication skills. They would definitely repeat the project, indicating their high degree of satisfaction. Those students who merely view the materials likewise have a very positive perspective of this type of initiative with their peers, and prefer a learning method using content videos created by their fellow students, to traditional learning with printed materials.

Another original finding is that recording the videos facilitates active learning among the students, in particular in the younger age groups. Furthermore, in terms of preparing the subject, it was found that the students that generated the digital material managed to fix and retain the content better and summarise the fundamental concepts of the exercise, representing a key additional benefit for the student-teachers. As for the distribution of the material, it would seem preferable to confine this to the educational context, although some creators suggested the use of YouTube as a social media platform, perhaps because it is more aligned with tutorials and support videos for a wide range of learning.

This type of educational experiment could have a notable role to play in improving hybrid and online teaching, if used properly. It could serve as a way of facilitating student motivation towards novel media, giving them greater responsibility and facilitating their involvement, or developing a problem-based focus, as indicated by Campbell et al. (2020). We furthermore agree with Belt and Lowenthal (2021) and with Rodríguez-Illera and Escofet-Roig (2006): the videos created by students lead to greater collaboration and skills development (in other words, technological capabilities) among the learners. This also promotes the construction of deeper mental models and understandings (Dyson & Frawley, 2018).

The pedagogical design of this experiment highlights a constructivist approach from the perception of the student, who takes on a more active role, even acting as teacher. And also in terms of interaction with other students, where we recognise the social nature of learning, as social constructivism theorises, postulating that learners build their understanding by interacting with a teacher and more advanced fellow students. This ties in with Vygotsky’s theory of the Zone of Proximal Development, in which “the level of potential development is determined through problem-solving under adult guidance or in collaboration with more capable peers” (Vygotsky, 1978, p. 86). In remote learning environments, it would be desirable to create the kind of Zone of Proximal Development that may arise through in-person teaching, or as Sangrà (2001) postulates, to promote learning networks, at all times placing students centre-stage in their learning, and in relation to their peers.

Size could constitute the main limitation of the research, although the sample does not differ from that used in other similar student-recorded video experiments (Dart et al., 2020). In any event, the original findings presented here could steer certain lines of development for learning in remote teaching. While also opening up new avenues of research for the better use of peer teaching and digital content in remote teaching.

## Data Availability

The datasets generated during and analyzed during the current study are not publicly available because individual privacy could be compromised, but are available from the corresponding author on reasonable request.
